# TNF-Stimulated Gene-6, Part of Extracellular Vesicles in Adipose Tissue-Derived Mesenchymal Stem Cell Concentrated Conditioned Medium, Affects Microglial Activity

**DOI:** 10.1007/s11481-025-10216-3

**Published:** 2025-05-29

**Authors:** Mohammad Shahadat Hossain, Pratheepa Kumari Rasiah, Amritha T. M. Seetharaman, Dulce Alvarado, Megan Luo, James A. Wohlschlegel, Mickey Pentecost, Rajashekhar Gangaraju

**Affiliations:** 1 Department of Ophthalmology, University of Tennessee Health Science Center, Memphis, TN USA; 2 Biomedical Engineering, Vanderbilt University, Vanderbilt Biophotonics Center, Nashville, TN USA; 3 Diadem Biotherapeutics, Inc, Torrance, CA USA; 4 Department of Biological Chemistry, University of California Los Angeles, Los Angeles, CA USA; 5 Department of Ophthalmology, Anatomy and Neurobiology, University of Tennessee Health Science Center, Memphis, TN USA

**Keywords:** Mesenchymal stem cells, Inflammation, Phagocytosis, Microglia, Exosome, CD44

## Abstract

Identifying the specific bioactive molecules produced by mesenchymal stem cells (MSCs) and the signaling pathways and cell types upon which they act is critical to developing MSC-based therapeutics for inflammatory diseases with high unmet needs. Our study aimed to investigate the impact of extracellular vesicle (EV)-derived TNF-Stimulated Gene-6 (TSG-6, from adipose tissue-derived mesenchymal stem cell concentrated conditioned medium, ASC-CCM or TSG-6 overexpression in ASC using ORF expression-ready clone) on microglia and its potential anti-inflammatory effects. EV but not non-vesicular secretome prepared by ultracentrifugation confirmed the expression of TSG-6 exclusively in the small EV (sEV) fraction. sEV ranged from 50–150 nm as determined by Zetasizer, demonstrated bilipid membrane evidenced by transmission electron microscopy, expressed positive exosomal (e.g. CD63) markers, and were endocytosed by BV2 cells confirmed by DiI fluorescently labeled exosomes. BV2 microglia cultured under serum-free conditions stimulated with TLR4 agonists (LPS and IFNγ) for 12 h in the presence of p-ASC-EV (sEV derived from ASC after cytokine stimulation) and TSG-6-ORF-EV significantly reduced nitrite release (*p* < 0.001), phagocytic activity (*p* < 0.001) and reduced CD44 expression (*p* < 0.05). CD44 knockdown in BV2 cells ablated TSG-6-ORF-EV mediated nitrite release, IL1β downregulation, and phagocytosis with TLR4 agonists. Our results revealed that under cytokine stimulation, the EV portion of ASC-CCM becomes enriched with TSG-6. Overexpressing TSG-6 in ASC leads to an increased concentration of TSG-6 in sEVs. This enriched EV fraction, containing TSG-6, regulates microglial dynamics through a feedback loop with CD44. EV-associated TSG-6 can influence immune cell behavior and signaling, mitigating excessive inflammation or immune dysfunction.

**Graphical Abstract:**

**Exosomal TSG-6 affects microglial activity**

This study examines TNF-Stimulated Gene-6 (TSG-6), a bioactive protein from mesenchymal stem cells that segregates into the small extracellular vesicle portion of the secretome and influences microglia behavior, reducing inflammation. Additionally, it highlights CD44’s role in mediating these effects and suggests that TSG-6-enriched exosomes suppress TLR signaling to regulate phagocytosis and inflammation.

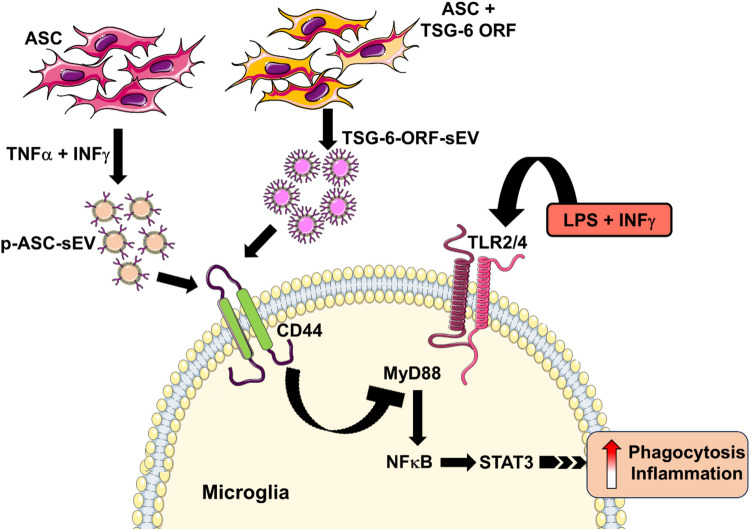

**Supplementary Information:**

The online version contains supplementary material available at 10.1007/s11481-025-10216-3.

## Introduction

Microglia, the resident immune cells of the central nervous system, play a crucial role in maintaining brain health and regulating neuroinflammation. Microglia are highly plastic and assume diverse phenotypes dependent on dynamic microenvironmental cues, such as inflammation and oxidative stress (Wu et al. 2021). Such chronic activation can contribute to the pathogenesis of various neurodegenerative and inflammatory diseases (Witcher et al. 2021; Na et al. 2014; Llorián-Salvador et al. 2024). Therefore, targeting microglial activation and promoting their anti-inflammatory phenotype holds immense therapeutic potential.

Mesenchymal stem cells (MSCs) have emerged as promising candidates for cell-based therapies due to their immunomodulatory and anti-inflammatory properties. Our previous research has shown that conditioned medium from adipose tissue-derived MSC (ASCs), which we termed ASC-CCM (adipose stem cell concentrated conditioned medium), has been shown to exert beneficial effects in various inflammatory models, including neuroinflammation (Jha, et al. 2018; Jha, et al. 2019; Jha et al. 2021; Elshaer et al. 2018). Importantly, we have identified TNF-Stimulated Gene-6 protein (TSG-6), a known anti-inflammatory molecule in MSCs, as the primary mediator of the therapeutic effect of ASC-CCM (Jha et al. 2019). TSG-6, a multifunctional protein involved in numerous biological processes, has recently gained attention for its anti-inflammatory and neuroprotective properties (Lesley et al. 2004; Choi et al. 2011; Liu et al. 2014). TSG-6 expression is upregulated in ASCs under inflammatory stimuli, and the lack of it abrogates the potential benefits, suggesting a pivotal role for TSG-6 in the beneficial effects of ASC-CCM (Jha et al. 2019).

ASC-CCM is a diverse and complex mixture of extracellular vesicles (EV) and soluble proteins. Recent studies have highlighted the role of sEVs, nanovesicles containing microRNAs, proteins, and lipids, as key mediators of cell-to-cell communication and intercellular signaling (O’Brien et al. 2020; Pascual et al. 2020; Couch et al. 2021; Tkach and Théry 2016). Recent studies demonstrate that TSG-6 is a cargo of MSC sEV (Jha et al. 2019; An et al. 2020; Jiang et al. 2020; Roura, et al. 2020). The association of TSG-6 with sEVs may enhance its bioactivity as the anchoring of therapeutic proteins on sEVs can increase their avidity for target receptors and tissue half-life relative to soluble recombinant molecules (Jafari et al. 2020; Mohammadi et al. 2021; Dooley et al. 2021; Staufer et al. 2022). Thus, reducing the complexity of ASC-CCM may be possible by refining the cellular product by segregating the sEV portion from the non-vesicular secretome.

This study aims to investigate the impact of EV-derived TSG-6 on microglial activation and its potential anti-inflammatory effects. We hypothesize that TSG-6 in ASC is an EV cargo and mitigates microglial inflammatory responses through specific interactions with CD44 receptors. We provide evidence that bioactive TSG-6 protein released from MSCs segregates into the sEV portion of the secretome. Additionally, we show that TSG-6 enriched sEVs act via the CD44 receptor to suppress TLR signaling in microglia and regulate phagocytosis and inflammation. Our results present an unrecognized superior therapeutic potential of TSG-6 enriched sEVs from ASC that are distinctly different from soluble TSG-6 or non-vesicular secretome.

## Materials and Methods

### ASC Culture and sEV Isolation

Human adipose derived mesenchymal stem cells (ASC) were purchased from ATCC (ASC52 telo, SCRC-4000) and cultured in low serum containing mesenchymal stem cell growth kit as per the manufacturer’s instructions as adherent cultures. ASC52 telo is an hTERT immortalized MSC line derived from the adipose tissue of a White female and extensively characterized for MSC phenotype (Choi et al. 2023; Wolbank et al. 2009; Mallard et al. 2023) and were not further characterized to avoid redundancy. Studies involving ASC were approved as an exempt study by UTHSC Institutional Review Board (16–04861-NHSR, 10/06/2016) and HRPO, US Army Medical Research and Materiel Command, in accordance with relevant guidelines and regulations following the tenets of the tenets of the Declaration of Helsinki. Cells were grown in T150 cm^2^ flasks as adherent cultures and maintained in a 37 °C incubator containing 5% CO_2_. Cells within the 5–15 passages were shown to exhibit the expected fibroblast-like morphology were used in the experiments. After reaching the confluency, cells were trypsinized (1X, TrypLE™ Express Enzyme) and seeded into T-75 flask at a density of 5 X 10^3^ cells/cm2 in MSC growth media. After 72 h post seeding, cells were washed twice with DPBS and proceeded with ASC-CCM preparation as described previously. Briefly, the growth medium was replaced with serum free MEM-alpha and cells were treated with 20 ng/ml TNFα and 10 ng/ml IFN$\gamma$. Cells untreated with cytokines served as controls (unprimed). After 24 h post treatment with cytokines, ASC were washed twice with DPBS and replaced with serum free MEM-alpha medium for another 24 h. After 24 h, the conditioned medium was collected and centrifuged at 300 g for 5 min; supernatants were collected and filtered through a 0.22 µm filter. The filtered conditioned medium was concentrated using an Amicon Ultra centrifugal filter (3 kDa, MilliporeSigma, Burlington, MA, USA), ultra-centrifuged (Sorvall WX ultracentrifuges, ThermoFisher Scientific) in 12 ml tubes at 30,000 rpm (~ 1,00,000 g) for 2 h at 4 oC. EVs were obtained from the bottom 200 $\mu$l and identified as p-ASC-EV (EVs derived from ASC after cytokine stimulation) and up-ASC-EV (unprimed native EVs). Carefully supernatants were removed to collect the EVs, protein estimation was obtained using Qbit Fluorimeter (ThermoFisher Scientific), size was determined by dynamic light scattering (DLS) method (Zetasizer, Malvern Panalytical Ltd, UK), bilipid membrane morphology by transmission electron microscopy, and EV markers utilizing either in-house slot blots or Exo-Check Exosome Antibody Array (System Biosciences, Palo Alto, CA). To label EVs, sEVs were incubated with Vybrant™ DiI Cell-Labeling Solution (Thermofisher) for 30 min at RT for 30 min protected from light. The unbound dye was removed by ultracentrifugation at ~ 1,00,000 g for 1 h. The labeled pellet was washed with DPBS to eliminate unbound dye and proceeded with uptake studies.

### TSG-6 Transient Transfection (Over Expression) of ASC and sEV Isolation

ASC cultured in mesenchymal stem cell growth kit were trypsinized and collected into serum free basal media were seeded into T-75 flask (~ 4 million cells in 10 ml media) and incubated with Lipofectamine™ RNAiMAX Transfection Reagent combined with 2$\mu \gamma$ of TSG6 Human Tagged ORF Clone (TNFAIP6, NM_007115, CAT#: RG206510, OriGene Technologies, Inc., Rockville, MD) for 5 min. Following this, mesenchymal stem cell growth kit media was added, and the cells were incubated in a 37 °C incubator containing 5% CO_2_. After 48 h of transfection, ASC was washed twice with DPBS and replaced with serum-free MEM-alpha medium for another 24 h. After 24 h, the conditioned medium was collected and proceeded to isolate sEVs by ultracentrifugation method as described above. The top 3 ml was considered as non-vesicular secretome (TSG-6-ORF-NV), while the bottom 200 $\mu$l was considered sEV fraction (TSG-6-ORF-EV).

As an additional method to purify and characterize sEVs, extracellular vesicles were isolated by size exclusion chromatography (SEC) followed by protein digestion and mass spectrometry analysis. Briefly, 50 ml of ASC-conditioned media with and without TSG-6 overexpression was concentrated and buffer-exchanged into Dulbecco’s phosphate-buffered saline (DPBS, Catlog#14190144, Thermofisher) using Amicon Ultra-15 centrifugal filters with a 10 kDa molecular weight cutoff (Catalog# UFC9010, Millipore Sigma). EVs were then isolated and purified from smaller biomolecules using qEV35 Gen2 SEC columns with an automated fraction collector (Izon Science, www.izon.com). A total of 0.5 mL of the concentrated medium was loaded onto the column, followed by the addition of DPBS. The first 3 mL, corresponding to the column void volume, was discarded. Subsequently, twelve 0.5 mL fractions were collected. Protein concentration in each fraction was measured using a Qubit protein assay and a Qubit fluorometer (ThermoFisher Scientific).

For downstream proteomic analysis, 10 µg of each sample was precipitated on ice for 1 h with trichloroacetic acid at a final concentration of 20%, centrifuged at 14,000 g for 30 min, washed and centrifuged at 14,000 g for 30 min twice with ice-cold acetone, air-dried, and stored at −20 °C until further processing. Protein samples were resuspended in 8 M urea in 100 mM Tris pH 8.5 and then digested using the protein aggregation capture method (Batth et al. 2019). The digested peptide solution was fractionated online using reversed phase chromatography and eluted directly into an Orbitrap Astral mass spectrometer (ThermoFisher) (Stewart et al. 2023). MS/MS spectra were collected using a data-independent analysis (DIA) acquisition method and subsequently analyzed using the DIA-NN algorithm (Guzman et al. 2024; Demichev et al. 2020). Peptide and protein identifications were filtered using an estimated false discovery rate of less than 1%. Pearson correlation coefficients were calculated for the comparison of each proteome’s spectral intensities. GOrilla was used for gene ontology (GO) enrichment analysis (GOrilla) (Eden et al. 2009). Proteomes were analyzed for common exosome markers, and the inclusion and exclusion markers were defined as described previously (Kugeratski et al. 2021). Z-score-transformed protein spectral intensities were used for heatmap visualization.

### Slot-Blot Analysis

Expression levels of EV markers (CD63, Alix and TSG6101) as well as TSG-6 were analyzed using Slot blot plate method (Catalog# 170–6542, Bio-slot SF, BIO-RAD) according to manufacturer instruction. Briefly, 20 µg of protein samples (40 µg of protein for Alix) were subjected to slot-blot analysis by loading samples onto a nitrocellulose membrane (Catalog# 1620161, BIO-RAD) with gentle vacuum. Antigen bound nitrocellulose membranes were then blocked with 3% BSA in 1X TBS (Tris-Buffer Saline, Catalog# BP2471-1, Fisher Bioreagents). Followed by washing with TBST (137 mM NaCl, 2.7 mM KCl, 20 mM Tris, pH7.4, plus 0.1% Tween-20), the membrane was incubated overnight with CD63 (1:500, Catalog#10628D, Thermofisher), Alix (1:500, Catalog#MA183977, Thermofisher), TSG101 (1:500, Catalog#MA123296, Thermofisher), GM130 (1:500, Catalog#MA535107, Thermofisher) and TSG6 (1: 500 R&D Systems, Cat.# AF2104). After washing with TBST, the membranes were incubated with HRP labeled secondary antibodies (1:1000, 2 h). After washing with TBST, the membranes were developed using SuperSignal™ West Pico PLUS Chemiluminescent Substrate (Thermofisher) and imaged with Odyssey infrared imager (LI-COR Biosciences) to visualize the protein bands. In studies utilizing Exo-Check™ Exosome Antibody Array, manufacturer’s instructions were followed.

### BV2 Cell Culture and Treatment Protocol

The mouse microglial cell line, BV2, was a kind gift from Professor Grace Sun, PhD, University of Missouri, Columbia, MO, USA. BV2 cells were cultured in DMEM containing 10% FBS and 1% penicillin–streptomycin. After reaching the confluency cells were trypsinized, counted and seeded at a density of 1 × 10^4^ cells/well of a 96-well plate. After 24 h media was aspirated, cells were washed with PBS and preincubated with unprimed (up-ASC-EV), p-ASC-EV (cytokine primed), TSG-6 overexpressed exosome (TSG-6-ORF-EV) and non-vesicular secretome (TSG-6-ORF-NV) fraction in serum free media (SFM) for 6 h. In some experiments, carrier free recombinant TSG-6 protein (Catalog # 2326-TS or Catalog # 2104-TS | R&D Systems, Inc) was utilized. Following this, cells were treated with LPS 100 ng/ml (Catalog# 10,602-HMAE, Sino Biological Inc.) and 10 ng/ml IFN$\gamma$ (Catalog# 315-05100UG, PEPROTECH) for 12 h. Cell supernatant and cell lysate were collected for Griess assay and RT-PCR, respectively. To determine if BV2 cells uptake sEVs, sEVs were pre-labeled with Vybrant™ DiI Cell-Labeling Solution (Thermofisher) and incubated with 1 × 10^4^ BV2 cells on 10 mm coverslips for 2 h and imaged them using Lionheart™ FX Automated Microscope (Biotek US., Winooski, VT, USA).

### CD44 Knockdown in BV2 Cells

To knockdown CD44 expression, BV2 cells were seeded at 1 × 10^4^ cells/well of a 96-well plate. After 24 h, cells were transfected for 48 h with 20 nM CD44 siRNA (Catalog#AM16708, Assay ID# 165,813, Life technologies corporation) with sense: 5`- CCAUGGACCAAAUGAAGUUTT-3´ and anti-sense: 5`- AACUUCAUUUGGUCCAUGGTG -3` nucleotides targeting the exon number 2 of transcript NM_001039150.1. Lipofectamine™ RNAiMAX Transfection Reagent alone served as transfection control. After 48 h, cells were washed with PBS and pre-incubated with sEVs or non-vesicular secretome in SFM for 12 h, then challenged with 100 ng/ml LPS and 10 ng/ml IFN$\gamma$ for 6 h. Cell supernatant and cell lysate were collected for Griess assay and RT-PCR, respectively.

### Nitric Oxide Release Assay

The nitrite amount in conditioned medium was assessed by Greiss Reagent System according to the manufacturer’s instructions (Promega, Madison, WI, USA) as described previously (Jha, et al. 2018).

### Phagocytosis Assay

BV2 cells were seeded at 2 × 10^4^ cells on 10 mm coverslips in a 24-well plate. After 24 h cells were washed with PBS and pre-incubated with unprimed (up-ASC-Exo), p-ASC-Exo (cytokine primed), TSG-6 overexpressed EV (TSG-6-ORF-EV) and non-vesicular secretome (TSG-6-ORF-NV) in SFM for 6 h, then challenged with LPS and IFN$\gamma$ for 4 h. In some experiments involving CD44 knockdown, BV2 cells were transfected with CD44 siRNA for 48 h prior to incubating the cells with sEVs or non-vesicular secretome. Following this, BV2 cells were washed with 1X PBS, incubated with Flurophores™-carboxylate red, 580/605 (Catalog# F8812, Invitrogen) for 2 h at 37 °C. After a brief wash to remove excess Fluorophores, cells were fixed with 4% buffered paraformaldehyde for 10 min, washed with PBS, permeabilized with 0.5% Triton in 1X PBS, labelled with actin phalloidin green in serum buffer (5 ml FBS, 45 ml 1X PBS, 200 $\mu$l of 5% Sodium azide). Finally, cells were washed with PBS, labeled with Hoechst (Catalog# H3570, Life Technologies, 1:1000 dilution) for 5 min. Coverslips were lifted from 24-well plate, mounted on slide and images were captured using Lionheart™ FX Automated Microscope (Biotek US., Winooski, VT, USA) with a 40 × objective. The number of Fluorospheres were quantitated in a blinded fashion from five images per treatment and averaged the number of fluorophores to the total number of cells counted to express phagocytosis index.

### RT-PCR

RNA was isolated from ASC after transfection of TSG-6 ORF clones or from BV2 cells after various treatments using NucleoSpin® RNA Plus kit (Macherey–Nagel GmbH, Takara Bio USA), following the manufacturer’s protocol. Subsequently, about 250 ng of total RNA from each sample was converted to cDNA using High-Capacity cDNA Reverse Transcription Kit (Thermo Fisher Scientific). The resulting cDNA sample served as a template for real-time qPCR using TaqMan probes or Sybergreen primers (Table 1) and accompanying Master Mix (Applied Biosystems, Foster City, CA). PCR amplification was carried out using Quantstudio3 (Applied Biosystems) system with cycle conditions of the initial cycle: 50 °C for 2 min, and initial denaturation at 95 °C for 15 s. This was followed by 40 cycles of denaturation at 95 °C for 15 s, and annealing/extension of 60 °C for 1 min. The expression levels of target gene transcripts were determined using 2 − DDCt method and normalized to 18S rRNA.

**Table 1:** Taqman probes and Sybr green PCR assay primers

Genes	Taqman Assay ID/Primer sequence	Reference Sequence
18S ribosomal RNA (18 s)	Mm04277571	NR_003278
Interleukin 1 beta (Il1β)	Mm00434228_m1	NM_008361.3
Cluster of Differentiation 44 (Cd44)	Mm01277161_m1	NM_009851
TNF-Stimulated Gene-6 (tsg6/TNFAIP6)	Hs00200180_m1	NM_007115
Cluster of Differentiation 68 (Cd68)	Mm03047343_m1	NM_001291058.1
apolipoprotein E (apoe)	Mm00437573_m1	AK010261
Cluster of Differentiation 73 (Cd73)	Forward: 5` -CAGTACCAGGGCACTATCTGG- 3` Reverse: 5` -AGTGGCCCCTTTGCTTTAAT- 3`	BC015940
Cluster of Differentiation 90 (Cd90)	Forward: 5` -ATGAACCTGGCCATCAGCA- 3` Reverse: 5` -GTGTGCTCAGGCACCCC- 3`	NM_006288
Cluster of Differentiation 105 (Cd105)	Forward: 5` -CCACTAGCCAGGTCTCGAAG- 3` Reverse: 5` -GATGCAGGAAGACACTGCTG- 3`	JN542540
Cluster of Differentiation 34 (Cd34)	Forward: 5` -TCTTGGGCATCACTGGCTATT-3` Reverse: 5` -GCCCAGCCTTTCTCCTGTG- 3`	AH000040
Cluster of Differentiation 45 (Cd45)	Forward: 5` -TGGTAAAAGCTCTACGCAAAGCT- 3` Reverse: 5` -AGGGTAGGTGCTGGCAATGA- 3`	Y00638
Elongation factor 1-alpha 1 (EF1α1)	Forward: 5`-AGATTGATCGCCGTTCTGGT- 3` Reverse: 5`-AGCAAAGCGACCCAAAGGT −3`	NM_001402

### Statistical Analysis

Data are presented as mean ± SEM from a single experiment repeated 2–3 times independently. For all quantitative experiments, statistical analyses were performed with one-way ANOVA followed by student t-tests. A *p*-value of < 0.05 was considered statistically significant. Data were analyzed for statistical interpretation using Microsoft Excel statistical package or Prism 9 software: GraphPad Software, La Jolla, CA.

## Results

### Cytokine Primed ASC-derived sEVs Suppress Microglia Activation in BV2 Cells

**Fig. 1:**
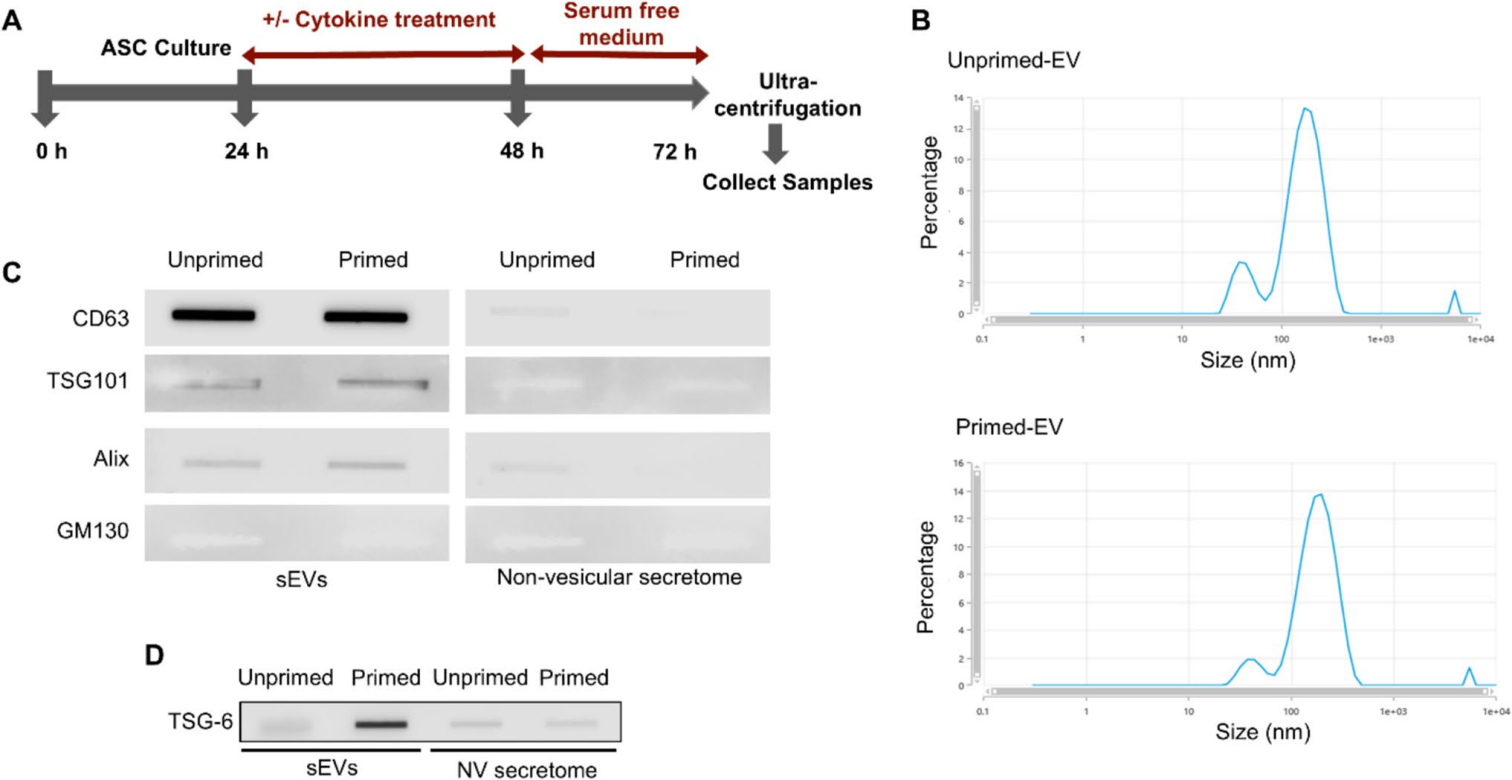
Characterization of ASC-CCM sEVs. (**A**) Timeline of ASC culture for cytokine stimulation and isolation of sEVs. (**B**). Particle size analysis by zeta sizer of unprimed (upEV) and primed (pEV) EV population. (**C**). Confirmation of sEV markers using semi-quantitative slot blot analysis, and (**D**). Slot bot analysis of TSG-6 expression in both EV and non-vesicular secretome. Data represent 3–4 independent experiments

Previously we have shown that cytokine-primed ASC-CCM enriched with TSG-6 inhibits the BV2 microglia and that the inhibition is lost when TSG-6 is knocked down with siRNA (Jha et al. 2019). In this study, following the same timeline that we established (Jha, et al. 2018) the conditioned medium from ASC that were primed with cytokines in serum free media were used to isolate sEVs by ultracentrifugation method (Fig. 1A). The sEVs derived from both primed and unprimed ASC were cup-shaped under scanning electron microscopy (Sup Fig. 1A) with diameters of 50–150 nm (Fig. 1B). Slot blot analysis demonstrated the typical exosomal surface markers CD63, CD81, ALIX, FLOT1, ICAM1, EpCam, ANXA5 and TSG101 and absence of a GM130 cis-Golgi marker confirming no cellular contamination (Fig. 1C and Sup Fig. 1B). It is noteworthy that the non-vesicular secretome portion of ASC conditioned medium is free of EV biomarkers (Fig. 1C and Sup Fig. 1B). Additionally, fluorescently labeled sEVs were successfully taken up by the BV2 microglia (Sup Fig. 1C) confirming the internalization of EVs into the target cells to release the cargo. Finally, slot-blot analysis confirmed the expression of TSG-6 only in the primed sEV but not in the unprimed and non-vesicular secretome portion of the ASC-CCM (Fig. 1D).

**Fig. 2:**
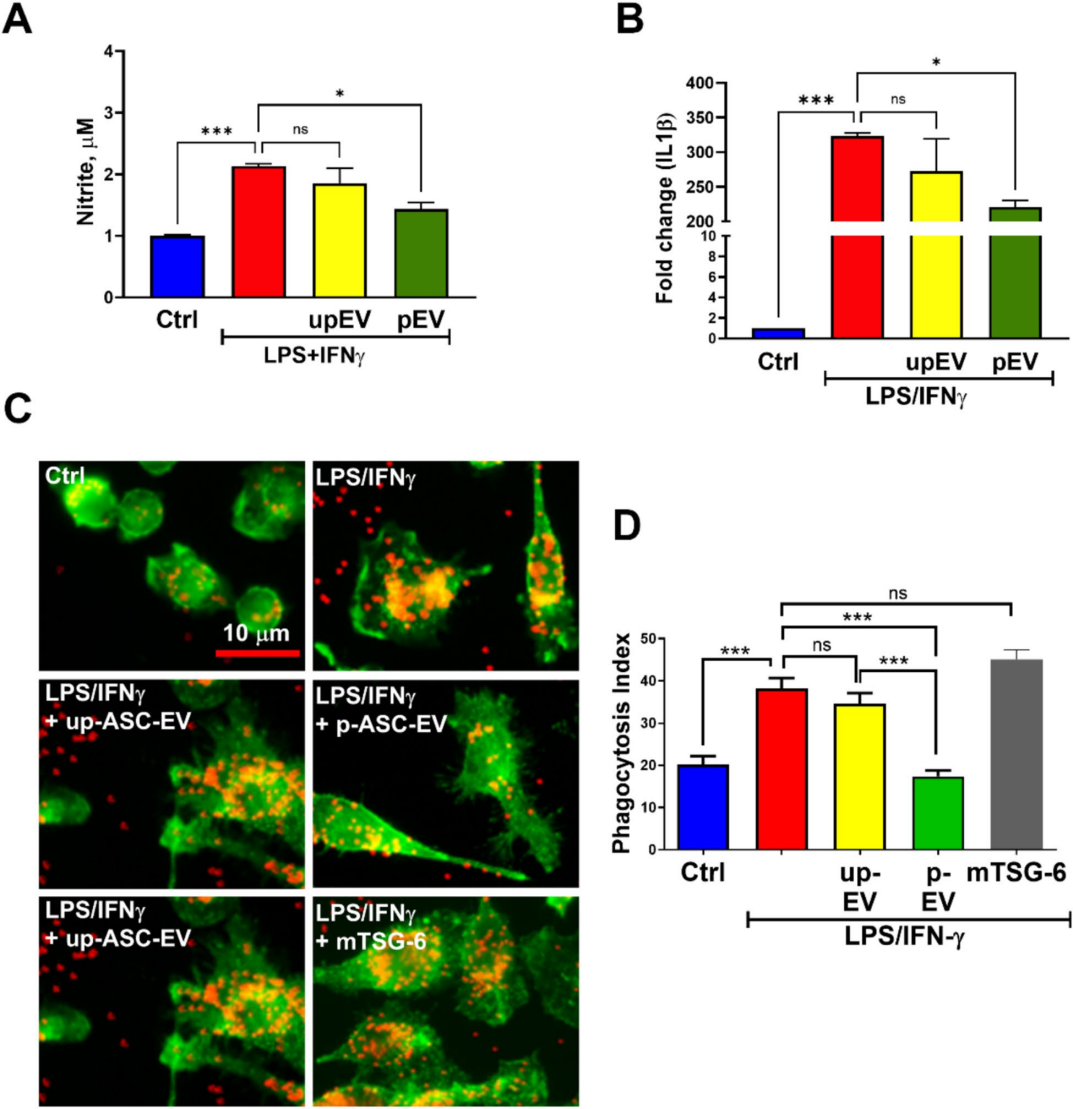
ASC-sEV enriched with TSG-6 suppress microglia activation. Primed sEVs suppresses LPS/IFN$\gamma$-stimulated (**A**) Nitrite release, (**B**), IL1β mRNA expression, and (**C** & **D**) phagocytosis in BV2 cells. Data represents *n* = 3/group. *p < 0.05; ** *p* < 0.01; ***p < 0.001; ns- not significant

Next, we investigated the efficacy of isolated primed ASC sEVs to suppress activation of BV2 microglia cell line and compared it to the unprimed sEVs. While primed sEVs could suppress the production of nitrite by LPS/IFN$\gamma$-treated BV2 cells, unprimed sEVs at the same total protein concentration (20 μg/ml) failed to suppress nitrite release (Fig. 2A). A similar observation is also made with changes in IL1β gene expression, a known microglia activation marker (Fig. 2B) and the ability to phagocytose fluorospheres (Fig. 2C&D). Interestingly, mouse recombinant TSG-6 failed to abrogate the increased phagocytosis in microglia, suggesting that EV-derived TSG-6 but not the soluble TSG-6 in the ASC provide the observed therapeutic effect.

### TSG-6 Overexpression in ASC-derived sEVs Suppresses Microglia Activation in BV2 Cells

**Fig. 3:**
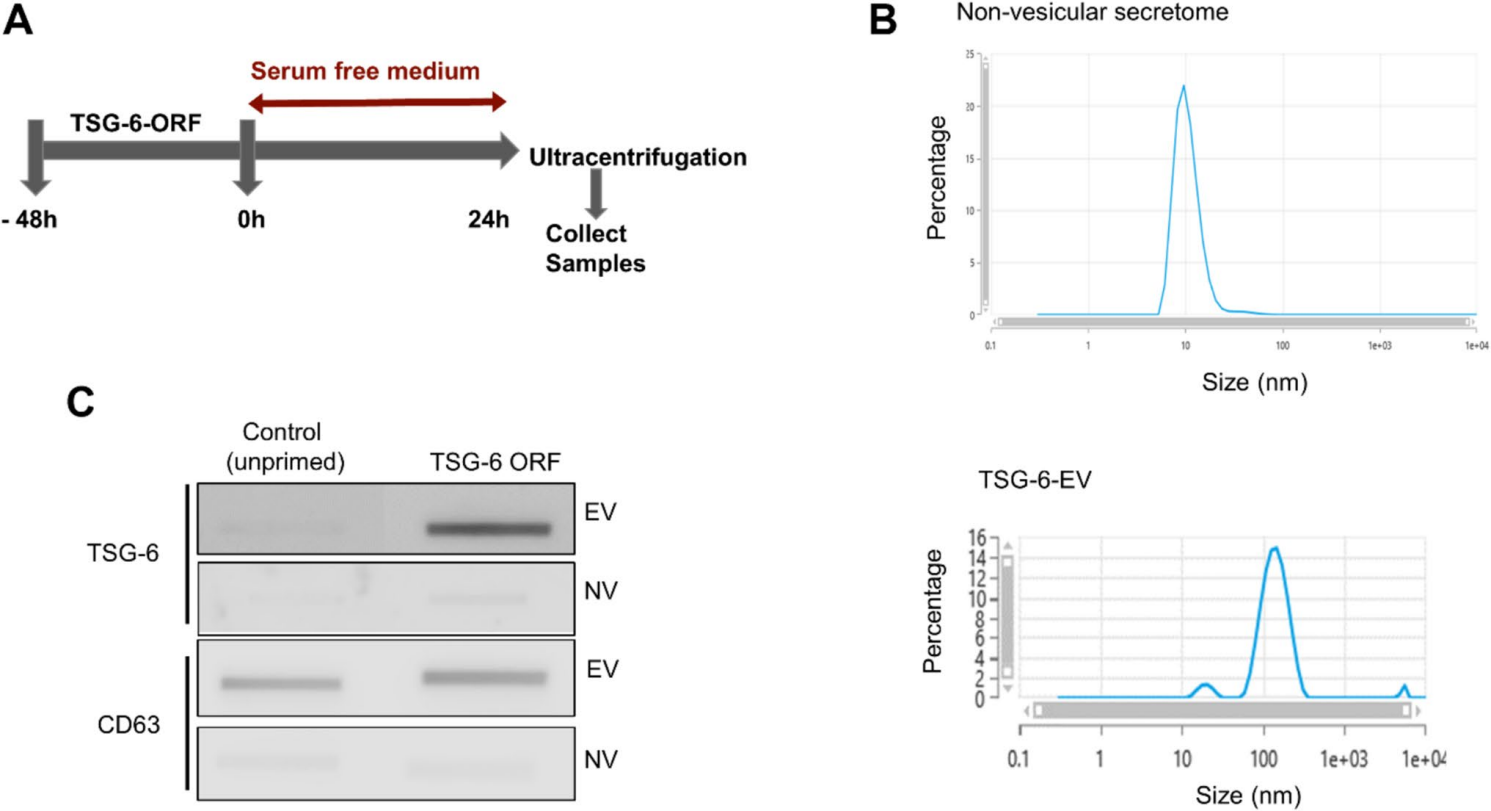
Characterization of ASC-TSG-6-ORF sEVs. (**A**) Timeline of ASC culture for TSG-6 overexpression and isolation of sEVs. (**B**). Particle size analysis by zeta sizer of non-vesicular secretome (NV) and sEV (EV) populations. (**C**). Confirmation of sEV marker CD63 and TSG-6 using semi-quantitative slot blot analysis. Data represent 3–4 independent experiments

Our data showed that priming is necessary to increase the TSG6 levels and the payload of TSG-6 is segregated in sEVs. Since the requirement for cytokine priming can result in product variability, we decided to test if TSG-6 could be transfected into ASCs to overexpress TSG-6 would also segregate into sEVs. To this end, we utilized commercially available open reading frame clone of TSG-6 to overexpress TSG-6 in ASC without cytokine stimulation (Fig. 3A). Since overexpression of TSG-6 might affect the integrity of ASC, we first confirmed the MSC markers, CD105, CD73, and CD90 are unaffected in ASC after 48 h post transfection with TSG-6 plasmid (Sup Fig. 2A). Following this, we confirmed a > tenfold increase in TSG-6 gene expression in transfected ASC (Sup Fig. 2B) and a > twofold increase in TSG-6 protein (Sup Fig. 2C). Next, we isolated the sEV (TSG-6-ORF-sEV) and non-vesicular secretome (TSG-6-ORF-NV) fraction from the ASC transfected with TSG-6-ORF clone following the ultracentrifugation method and re-confirmed them using SEC method. As expected, TSG-6-ORF-sEV prepared with ultracentrifugation method were within the expected size of 50–150 nm and are within the < 200 nm size to qualify as sEV per the MISEV guidelines (Welsh et al. 2024) (Fig. 3B) and expressed EV associated CD63 (Fig. 3C), while the non-vesicular secretome was below 30 nm in size and lacked the expression of CD63 (Fig. 3B&C). Interestingly, similar to cytokine-stimulated ASC, over-expression of TSG-6 in ASC resulted in the enrichment of TSG-6 in the sEVs (Fig. 3C).

**Fig. 4:**
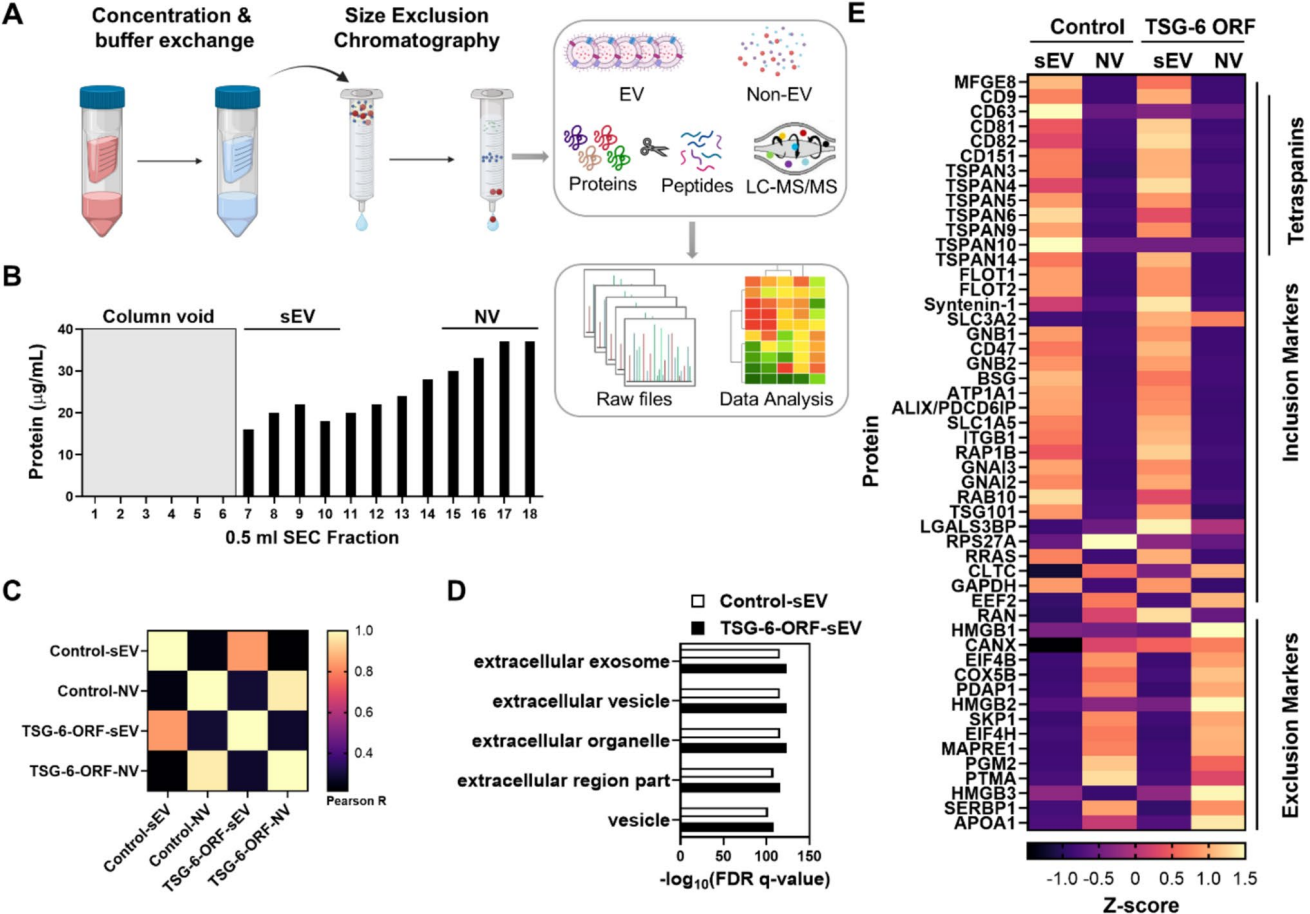
Size exclusion chromatography and proteomic analysis of ASC-TSG-6-ORF sEVs. (**A**) Scheme of purification of sEVs using ultrafiltration followed by SEC and quantitative proteomics. (**B**). Total protein measurements in collected SEC fractions. (**C**) Pearson correlation matrix for Control- and TSG-6-ORF sEV and NV proteomes. (**D**) Top five cellular compartment gene ontology enrichment terms for Control-sEV and TSG-6-ORF-sEV proteomes. (**E**) Heatmap of z-score standardized peptide intensities for exosome inclusion and exclusion protein markers within Control- and TSG-6-ORF sEV and NV proteomes. Data is from a single experiment

As an additional method to identify and characterize sEVs in the ASC secretome, we subjected SEC-isolated sEVs and NV secretome to shotgun proteomic analysis (Fig. 4A). The first four fractions immediately following the column void volume were pooled and designated as the small extracellular vesicle (sEV) sample. The final four fractions were pooled and designated as the non-vesicle (NV) sample (Fig. 4B). The Control-sEV and TSG-6-ORF-sEV proteomes showed high similarity to one another and low similarity to NV proteomes (Fig. 4C). The most highly enriched cellular component gene ontology terms for the sEV proteomes were extracellular exosomes, extracellular vesicle, extracellular organelle, extracellular region part and vesicle (Fig. 4D). Correspondingly, sEV proteomes were enriched for exosome inclusion marker proteins compared to NV proteomes, while NV proteomes were enriched for exosome exclusion protein markers compared to sEV proteomes (Fig. 4E and Supplemental Information) (Kugeratski et al. 2021). Taken together with the ultracentrifugation method, sEVs used in the study were positive for a variety of tetraspanins such as CD9, CD63, CD81, Alix and Syntenin-1and lacked contaminating non-vesicular proteins such as GM130, COX5B, SERBP1 and APOA1 (Kugeratski et al. 2021).

**Fig. 5:**
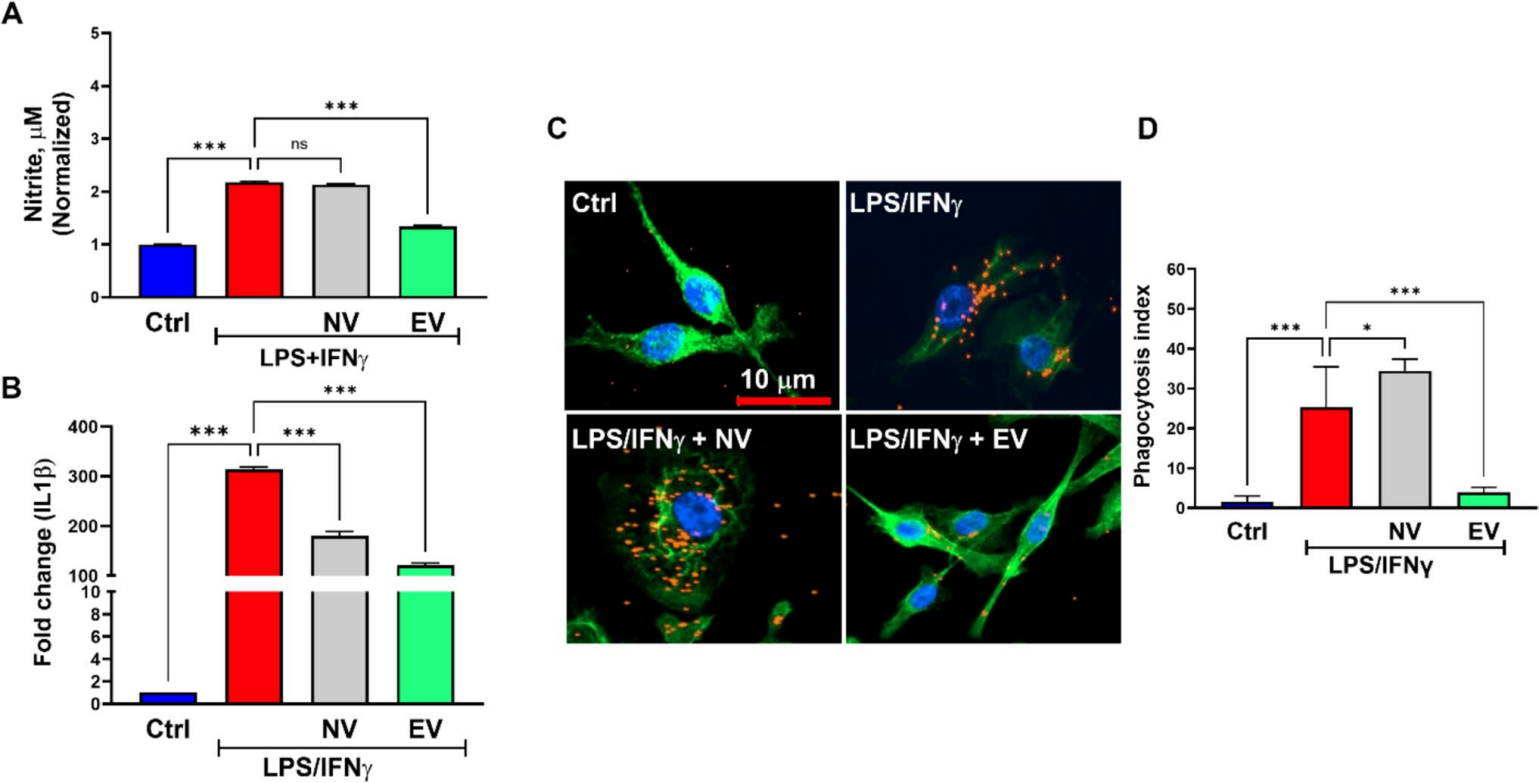
Over expression of TSG-6 in ASC enriched with TSG-6 in EV suppress microglia activation. EV fraction but not non-vesicular secretome (NV) derived from ASC transfected with TSG-6 ORF plasmid suppresses LPS/IFN$\gamma$- induced (**A**) Nitrite release, (**B**), IL1β mRNA expression, and (**C**) phagocytosis in BV2 cells. Data represents *n* = 3/group. *p < 0.05; ** *p* < 0.01; ***p < 0.001; ***p < 0.001; ns- not significant

Next, we investigated the efficacy of TSG-6-ORF-EV in suppressing activation of BV2 microglia cell line and compared it to the TSG-6-ORF-NV. While TSG-6-ORF-EV could suppress the production of nitrite by LPS/IFN$\gamma$-treated BV2 cells, the non-vesicular secretome at the same total protein concentration (20 μg/ml) failed to suppress nitrite release (Fig. 5A). A similar observation is also made with changes in IL1β gene expression (Fig. 5B) and the ability to phagocytose fluorospheres (Fig. 5C&D). Our results confirm that EV delivery of TSG-6 but not the non-vesicular secretome in ASC mitigates excessive inflammation and microglia dysfunction.

### EV-derived TSG-6 Suppresses Microglia Activation via Cognate Receptor CD44

**Fig. 6:**
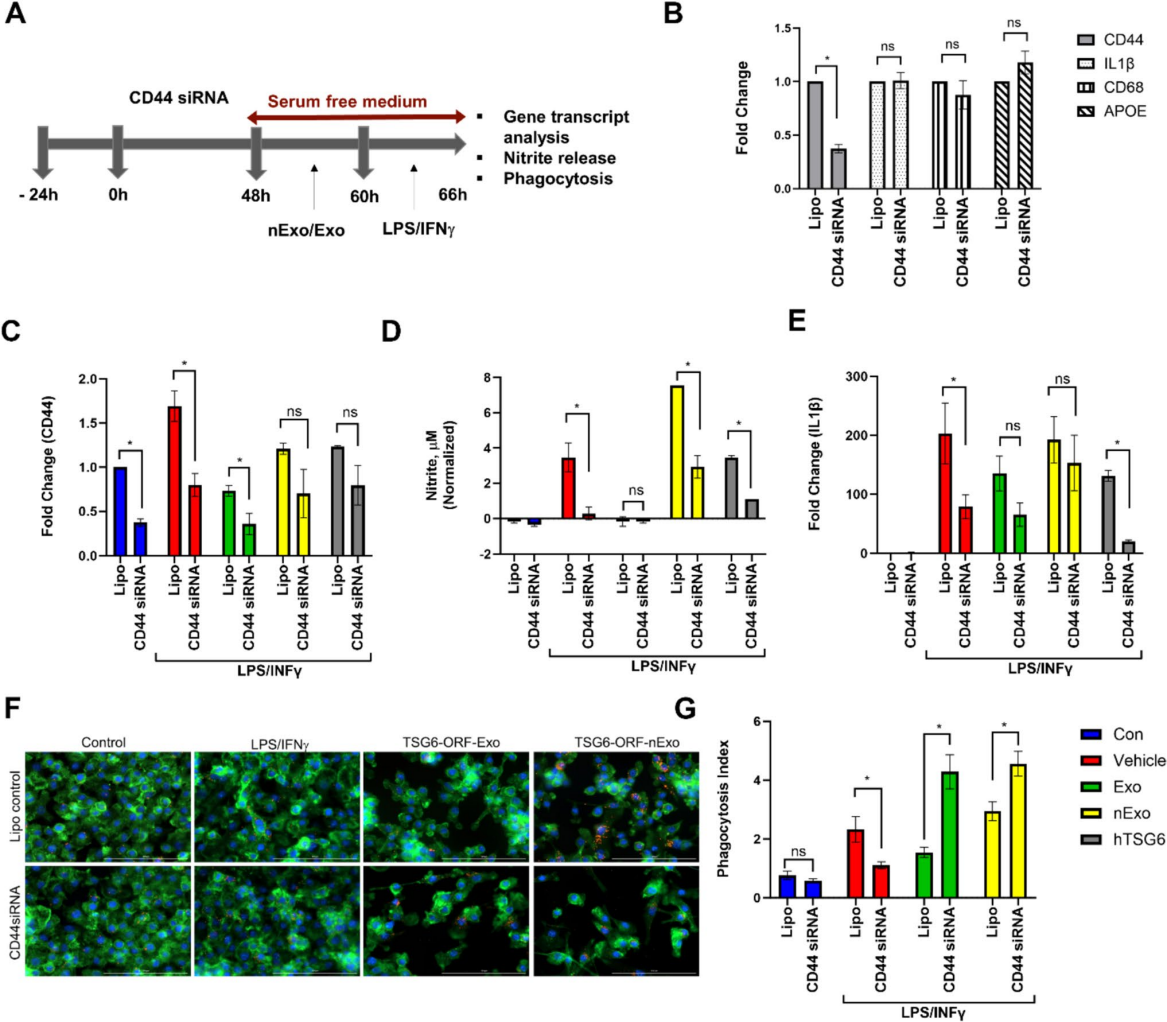
EV-derived TSG-6 suppresses microglia activation via cognate receptor CD44. (**A**) Timeline of BV2 microglia culture and CD44 knockdown. (**B**). CD44 mRNA expression significantly reduced in sEVs but not non-vesicular secretome. (**C**). TSG-6 EV failed to suppress LPS/IFN$\gamma$-stimulated (C) Nitrite release, (**D**), IL1β mRNA expression, and (E&F) phagocytosis in BV2 cells. Data represents *n* = 3/group. *p < 0.05; ** *p* < 0.01; ***p < 0.001; ns- not significant

Previous studies have found that TSG-6 interacts with CD44 to elicit its cell signaling (Liu et al. 2014; Wang et al. 2020). In this study, we wanted to assess if CD44 is necessary for TSG-6 EV-mediated therapeutic activity in BV2 microglia. To this end, we utilized the siRNA approach to knockdown CD44 in activated BV2 cells in the presence or absence of TSG-6-ORF-NV and TSG-6-ORF-EV (Fig. 6A). In some experiments, carrier free recombinant TSG-6 protein was utilized to understand the relationship between exosome derived TSG-6 to soluble TSG-6 on the CD44 expression. First, we confirmed the specificity of CD44 knockdown in BV2 microglia with no changes in other microglial markers (Fig. 6B). As expected, CD44 expression increased in activated microglia when challenged with LPS/IFN$\gamma$. On the other hand, knockdown with CD44 siRNA showed a robust decrease in CD44 expression in control as well as in activated BV2 microglia, confirming the siRNA knockdown approach (Fig. 6C). While TSG-6-ORF-NV and soluble TSG-6 failed to decrease CD44 expression in activated BV2 microglia, significantly suppressed nitrite release and IL1β gene expression. On the other hand, though the presence of TSG-6-ORF-EV further reduced the CD44 expression (Fig. 6C), it failed to decrease the nitrite levels (Fig. 6D), IL1β gene expression (Fig. 6E) and significantly increased the rate of phagocytosis (Fig. 6F&G) in activated microglia in the cells knockdown for CD44. This suggests that CD44 is necessary for TSG-6 sEVs to function to elicit their anti-inflammatory activities.

## Discussion

Our study provides compelling evidence for the anti-inflammatory role of EV-derived TSG-6 in modulating microglial activation. We demonstrated that TSG-6 specifically enriches the sEV fraction of adipose tissue-derived mesenchymal stem cell concentrated conditioned medium (ASC-CCM) under cytokine stimulation or TSG-6 overexpression utilizing ORF plasmid. This enrichment significantly enhances its anti-inflammatory effects on BV2 microglia, as evidenced by reduced nitrite release, CD44 expression, inflammation, and phagocytosis. These findings suggest that TSG-6 acts as a key bioactive cargo within these sEVs, directing their immunomodulatory function.

The anti-inflammatory properties of TSG-6 are documented in numerous models of inflammation and injury (An et al. 2020; Jiang et al. 2020; Yang et al. 2020; Day and Milner 2019; Watanabe et al. 2013; Kim et al. 2015). TSG-6 is not expressed under normal conditions but is rapidly induced in many cell types in response to proinflammatory mediators such as TNF-α, interleukin-1, interferon $\gamma$ (IFN$\gamma$), and Toll-like receptor (TLR) agonist lipopolysaccharide (LPS) (Milner and Day 2003). We have shown that IFN$\gamma$ synergizes with TNF-α for TSG-6 expression in ASC and that TSG-6 containing ASC-CCM from cytokine-primed ASCs has a greater capacity to suppress the activation of cultured microglia and therapeutic effect in several models of retinal inflammatory diseases (Jha et al. 2018; Jha et al. 2019; Jha et al. 2021; Elshaer et al. 2018). Moreover, we showed that siRNA-mediated depletion of TSG-6 in cytokine primed ASC-CCM abrogates the protective effect, implicating TSG-6 as a therapeutic protein (Jha et al. 2019).

A primary signaling target of TSG-6 is the adhesion molecule CD44 that is upregulated on immune cells and parenchymal cells during inflammation (Choi et al. 2011). Association of TSG-6 with hyaluronic acid (HA) enhances the CD44-HA interaction and modulates inflammatory signaling (Choi et al. 2011). Suppression of NFκB signaling downstream of CD44 appears to be a primary mechanism by which TSG-6 suppresses macrophage and microglial cell activation (Lesley et al. 2004; Choi et al. 2011; Liu et al. 2014). In this regard, we have shown previously that TSG-6 is necessary to suppress STAT3, a downstream transcription factor of NFκB signaling (Jha et al. 2019). In this study, we have identified CD44 as a cognate receptor for exosome derived TSG-6. The abrogation of TSG-6-mediated anti-inflammatory effects upon CD44 knockdown underlies its crucial role in this process. A similar finding was noted previously in BV2 microglia utilizing soluble TSG-6 (Liu et al. 2014), although we were unable to confirm a direct correlation between soluble TSG-6 and CD44. Although we have not studied the direct interaction between TSG-6 and CD44 in this study, a previous study in other cell types has shown such direct interaction (Wang et al. 2020). Our studies suggest that CD44 is necessary and sufficient for TSG-6 sEV-mediated suppression of pro-inflammatory microglia activation as determined by nitrite production and IL1β gene expression; the non-vesicular secretome and soluble TSG-6 effects are independent of CD44. On the other hand, TSG-6 sEVs and non-vesicular secretome are highly dependent on CD44 in activated BV2 microglia to suppress phagocytosis. It may be a possibility that EV derived TSG-6 is associated with other proteins thus may function differently. Another possibility is that owing to increased receptor avidity, EV tethered TSG-6 likely have greater activation of receptor signaling than comparable concentrations of soluble TSG-6. Future research is necessary to explore the specific co-purifying proteins in the TSG-6 sEVs and their signaling pathways downstream of CD44 in activated microglia downstream of EV-derived TSG-6 signaling.

Our study has significant implications for therapeutic development in various inflammatory diseases. The ability of ASC-CCM sEVs, particularly those enriched with TSG-6, to modulate microglial activation suggests their potential as therapeutic agents for neurodegenerative diseases and other conditions with chronic inflammation. Our studies to obtain TSG-6 in sEVs in ASC without cytokine stimulation should provide a simplified way to generate the therapeutic. This, in turn, likely helps reduce the complexity of ASC-CCM for translational studies by helping to establish a consistent, scalable cell source and the development of robust cGMP-compliant upstream and downstream manufacturing processes. Further investigations are needed to validate our findings in in vivo models and refine the isolation and delivery strategies for EV-associated TSG-6.

We readily recognize the limitations of this study. Techniques like ultracentrifugation are poorly scalable and DLS measurements has limited accuracy for small particles. Use of alternate purification and characterization methods are crucial for validating the results. The gene expression of IL1β and CD44 may not accurately represent protein expression or capture the functional role of these proteins at cellular level. Simplified in vitro model using cultured microglia cannot not fully replicate in vivo environment or complex disease pathology. While the study focuses on TSG-6, other bioactive molecules in the EVs might synergize with or antagonize the observed effects. Although the study identifies a feedback loop involving CD44, the detailed molecular mechanisms underlying this interaction remain unclear. Further research is needed to elucidate these pathways.

In conclusion, our study unveils a novel anti-inflammatory pathway mediated by exosomal-derived TSG-6 from ASC and its interaction with CD44 in microglia. These findings contribute to a deeper understanding of the immunomodulatory properties of ASC-CCM and pave the way for future translational applications in tackling chronic inflammatory diseases.

## Supplementary Information

Below is the link to the electronic supplementary material.

Supplementary file1 Sup Figure 1: Characterization of cytokine-primed ASC-CCM sEVs.(A) EV morphology by transmission electron microscopy, (B) confirmation of EV marker using semi-quantitative Exo-check exosome antibody array. (C) DiI fluorescently labeled sEVs endocytosed by BV2 cells. Sup Figure 2: Characterization of ASC after TSG-6 overexpression.(A). Normalized fold change of MSC markers with increasing concentrations of TSG-6-ORF plasmids as compared to non-transfected native ASC. (B). Fold change TSG-6 mRNA expression in transfected cells. (C). Increased protein levels of TSG-6 in transfected cells. (DOCX 1297 KB)

Supplementary file2 (XLSX 20 KB)

Supplementary file3 (PDF 1407 KB)

## Data Availability

No datasets were generated or analysed during the current study.

## Abbreviations

ASC: Adipose stem/stromal cells
ASC-CCM: Adipose tissue-derived mesenchymal stem cell concentrated conditioned medium
CD44: Cluster of differentiation 44
CD63: Cluster of differentiation 63
EV: Extracellular vesicle
sEV: Small EV
Exo: Exosomes
IFNγ: Interferon gamma
LPS: Lipopolysaccharide
MSC: Mesenchymal stem/stromal cells
ORF: Open reading frame
p-ASC-EV: Cytokine primed-ASC-extracellular vesicles
TLR4: Toll like receptor-4
TSG-6: TNF-Stimulated Gene-6
up-ASC-EV: unprimed-ASC-EVs
NV: Non-vesicular secretome
