# Supplementary figures and images for: TNF-Stimulated Gene-6, Part of Extracellular Vesicles in Adipose Tissue-Derived Mesenchymal Stem Cell Concentrated Conditioned Medium, Affects Microglial Activity
Source: J Neuroimmune Pharmacol. 2025 May 29;20(1):60. doi: 10.1007/s11481-025-10216-3 (PMC12122589; doi:10.1007/s11481-025-10216-3)

**Supplemental Information**

**Supplemental Figure 1**

**
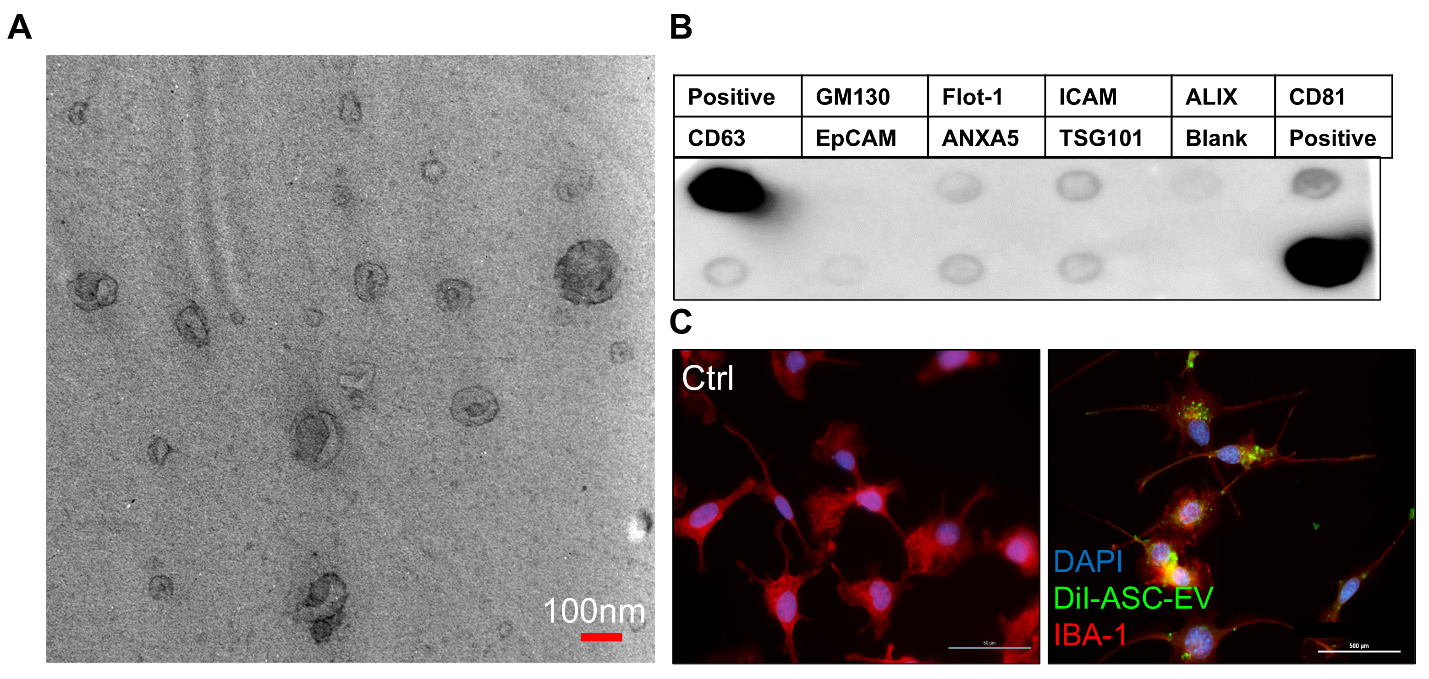
**

**Supplemental Figure 2**

**
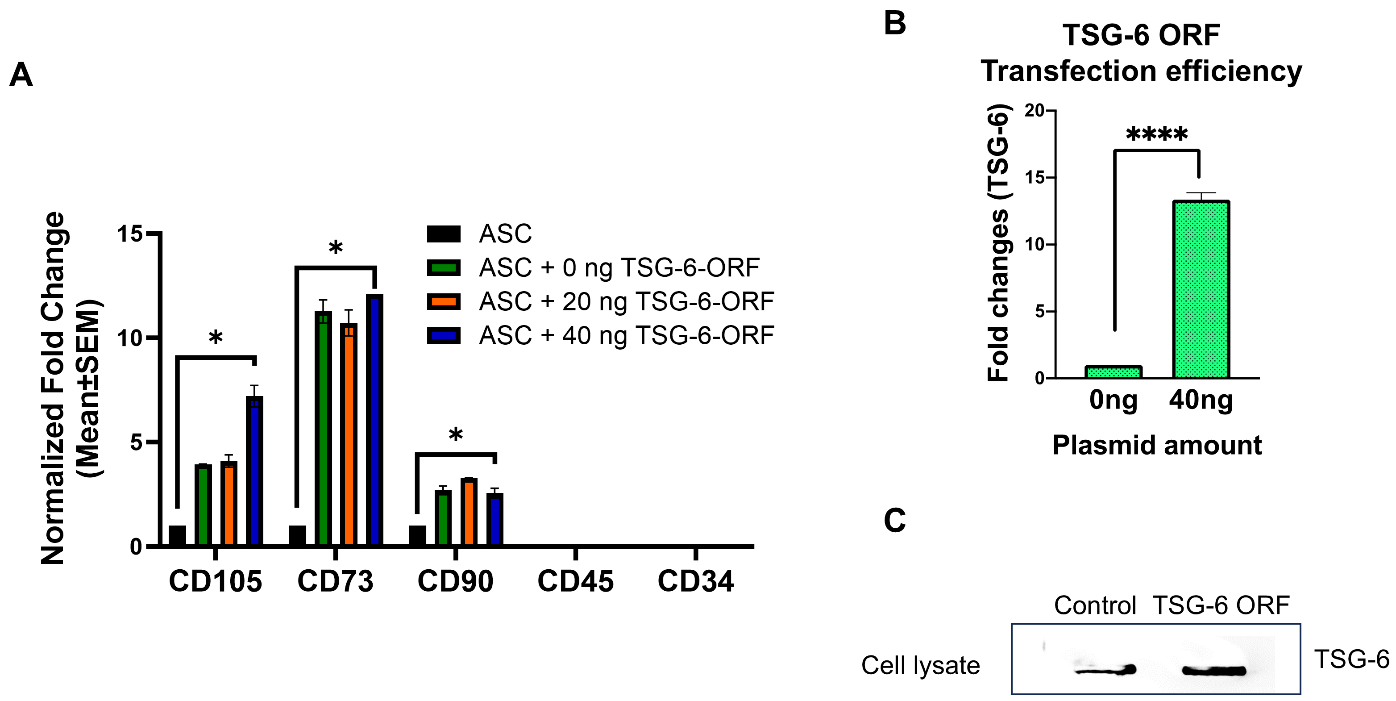
**

Supplement: Supplementary file 1 — Supplementary file1 Sup Figure 1: Characterization of cytokine-primed ASC-CCM sEVs.(A) EV morphology by transmission electron microscopy, (B) confirmation of EV marker using semi-quantitative Exo-check exosome antibody array. (C) DiI fluorescently labeled sEVs endocytosed by BV2 cells. Sup Figure 2: Characterization of ASC after TSG-6 overexpression.(A). Normalized fold change of MSC markers with increasing concentrations of TSG-6-ORF plasmids as compared to non-transfected native ASC. (B). Fold change TSG-6 mRNA expression in transfected cells. (C). Increased protein levels of TSG-6 in transfected cells. (DOCX 1297 KB) [file 11481_2025_10216_MOESM1_ESM.docx]
