# Supplementary material for: TNF-Stimulated Gene-6, Part of Extracellular Vesicles in Adipose Tissue-Derived Mesenchymal Stem Cell Concentrated Conditioned Medium, Affects Microglial Activity
Source: J Neuroimmune Pharmacol. 2025 May 29;20(1):60. doi: 10.1007/s11481-025-10216-3 (PMC12122589; doi:10.1007/s11481-025-10216-3)
Supplement: Supplementary file 3 — Supplementary file3 (PDF 1407 KB) [file 11481_2025_10216_MOESM3_ESM.pdf]

TNF-Stimulated Gene-6, part of extracellular vesicles in adipose tissue-derived mesenchymal stem cell concentrated conditioned medium, affects microglial activity

Mohammad Shahadat Hossain, Pratheepa K Rasiah, Amritha TM Seetharaman, Dulce Alvarado, Megan Luo, James A Wohlschlegel, Mickey Pentecost, Rajashekhar Gangaraju.

Raw data

Figure 1:

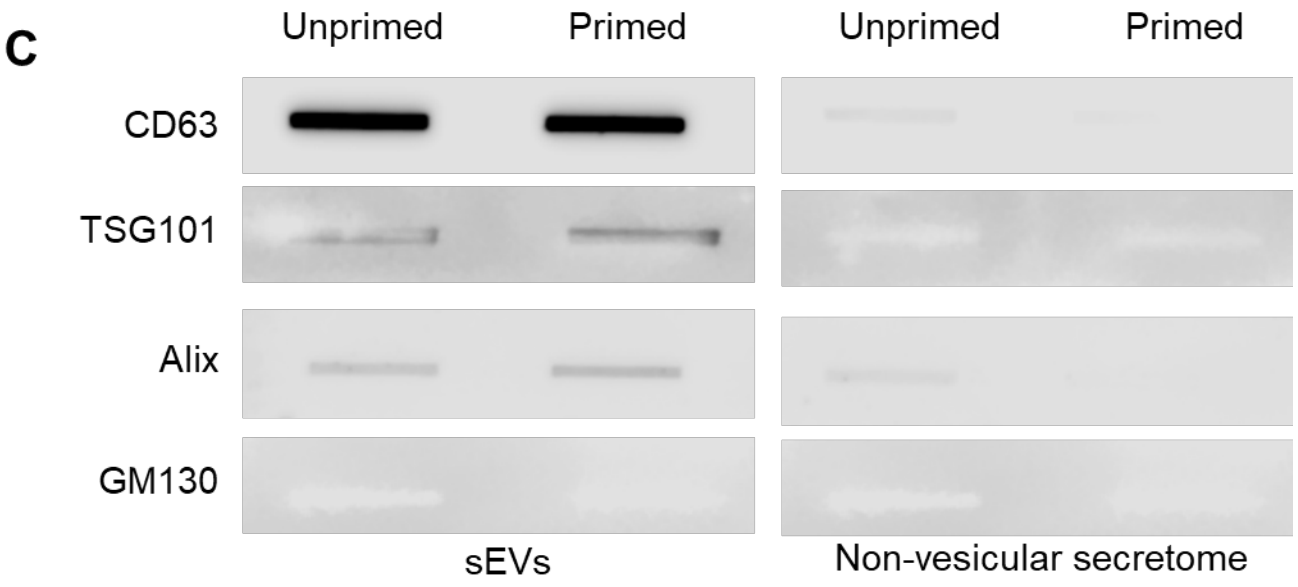
C
Unprimed
Primed
Unprimed
Primed
CD63
TSG101
Alix
GM130
sEVs
Non-vesicular secretome

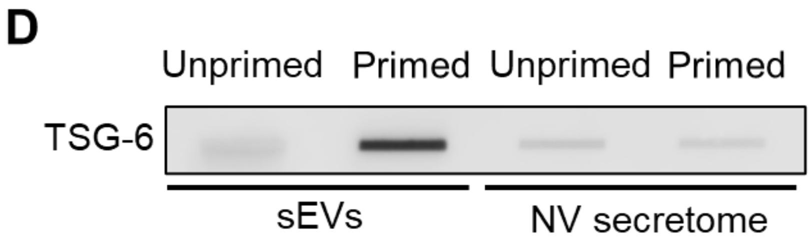
D
Unprimed
Primed
Unprimed
Primed
TSG-6
sEVs
NV secretome

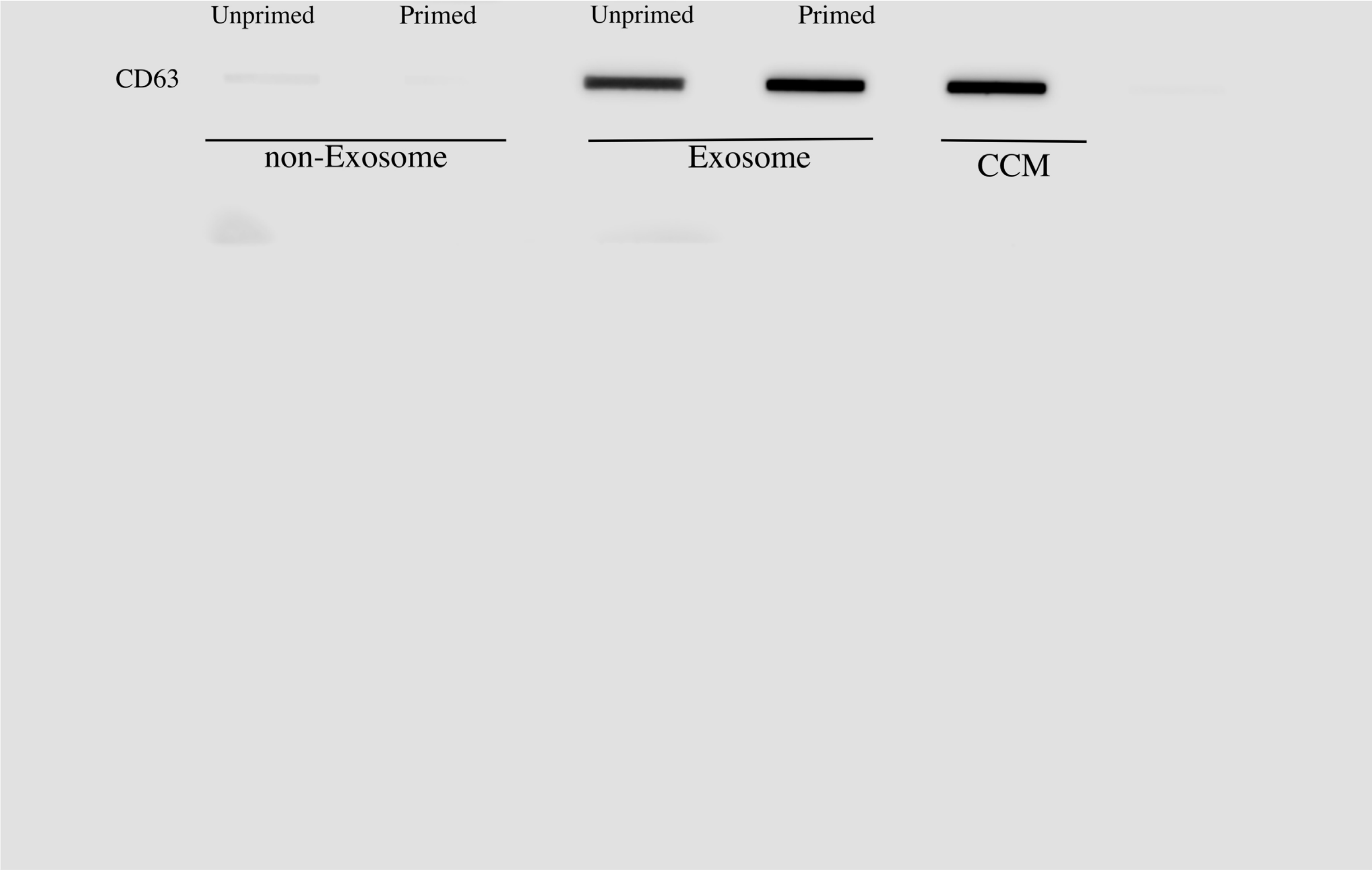
Unprimed
Primed
Unprimed
Primed
CD63
non-Exosome
Exosome
CCM

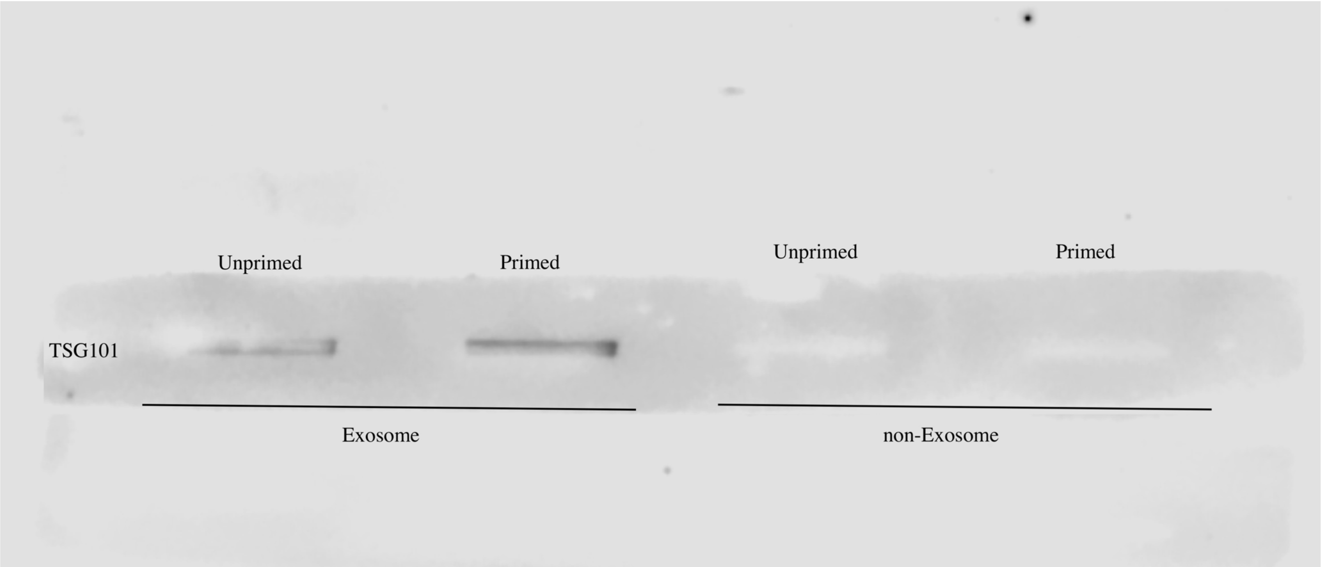
Unprimed
Primed
Unprimed
Primed
TSG101
Exosome
non-Exosome

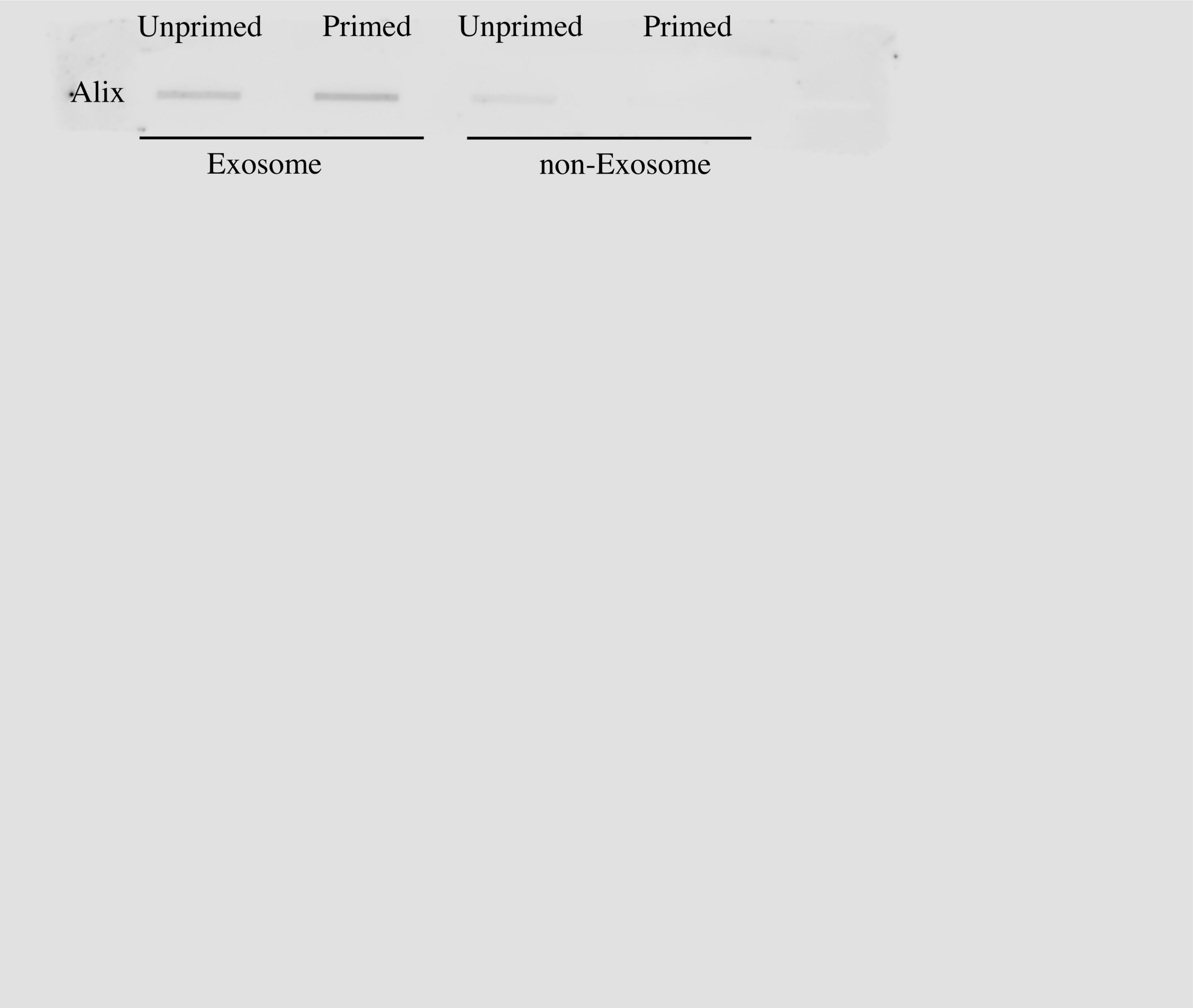
Unprimed
Primed
Unprimed
Primed
Alix
Exosome
non-Exosome

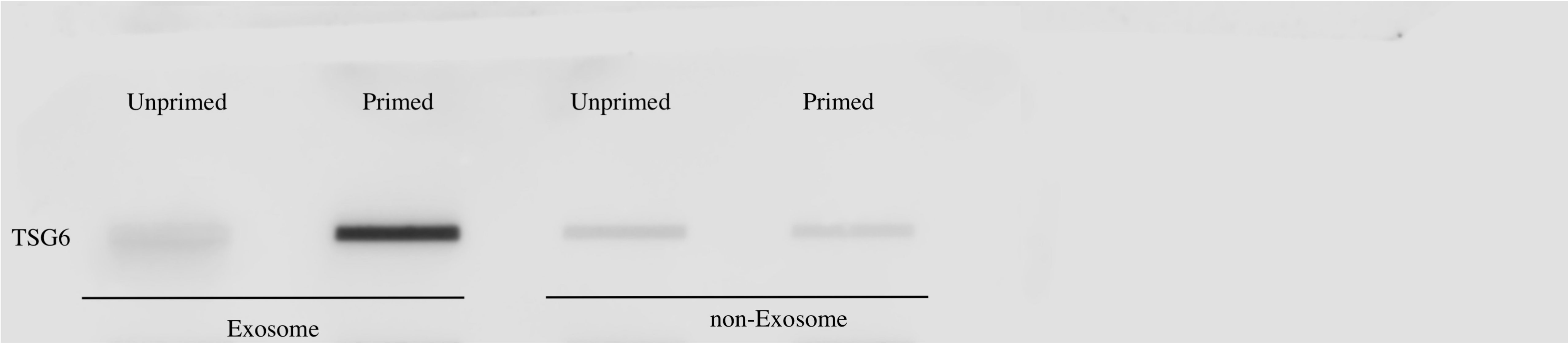
Unprimed
Primed
Unprimed
Primed
TSG6
Exosome
non-Exosome

Figure 3:

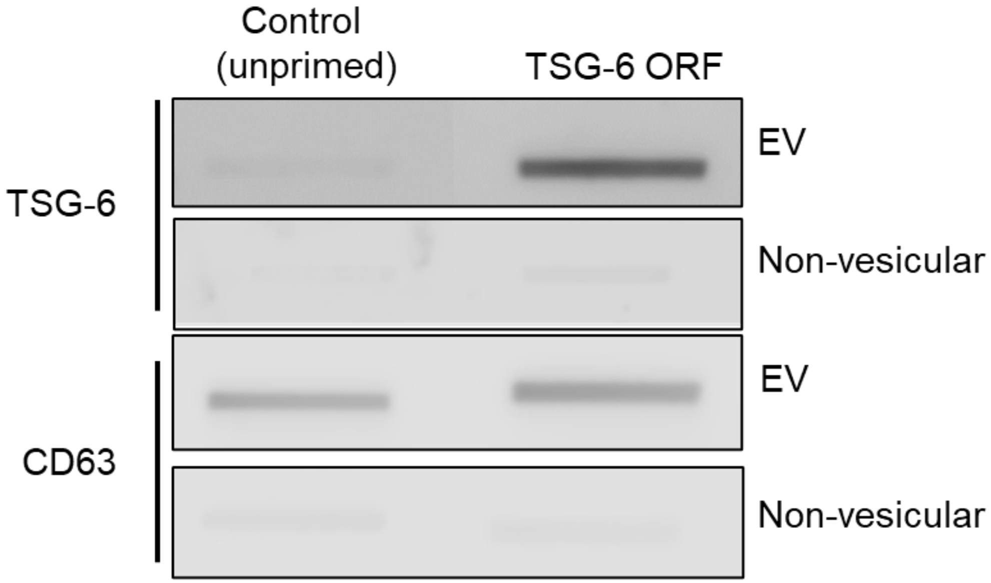
Control
(unprimed)
TSG-6 ORF
TSG-6
EV
Non-vesicular
CD63
EV
Non-vesicular

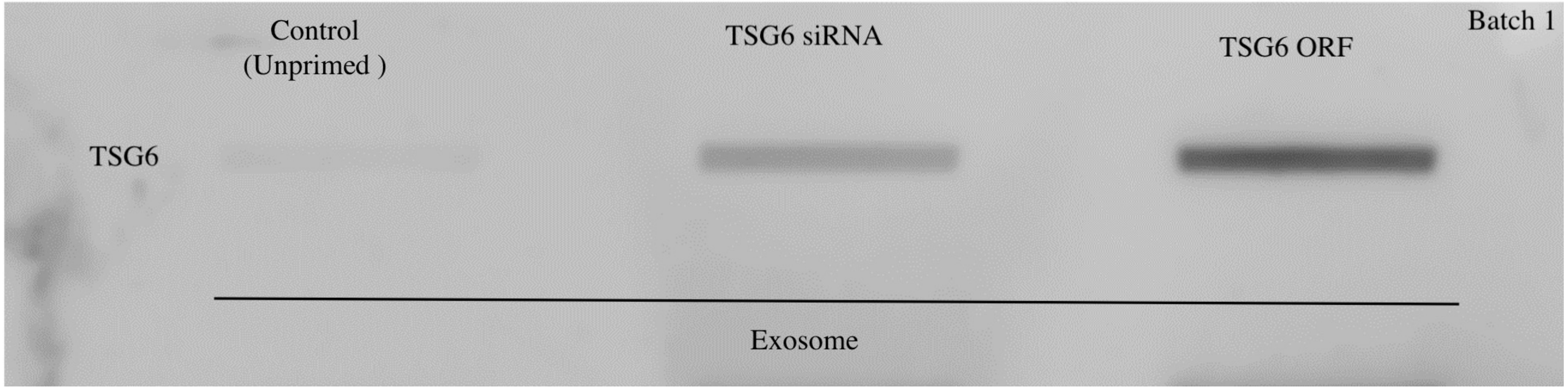
Batch 1
Control
(Unprimed)
TSG6 siRNA
TSG6 ORF
TSG6
Exosome

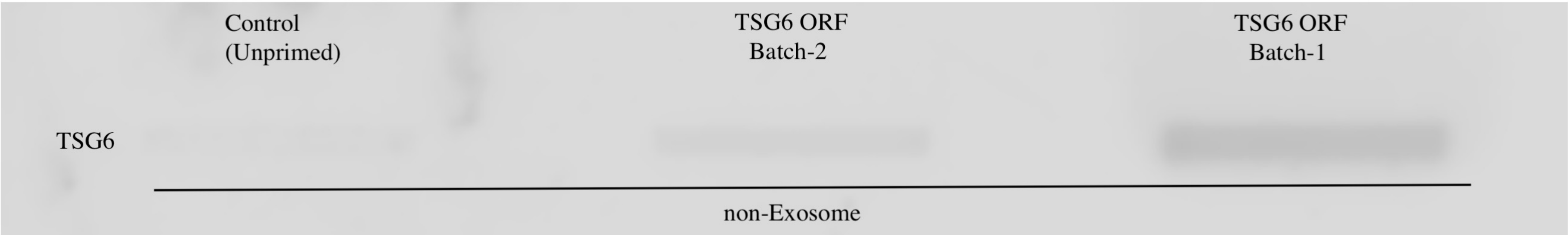
Control
(Unprimed)
TSG6 ORF
Batch-2
TSG6 ORF
Batch-1
TSG6
non-Exosome

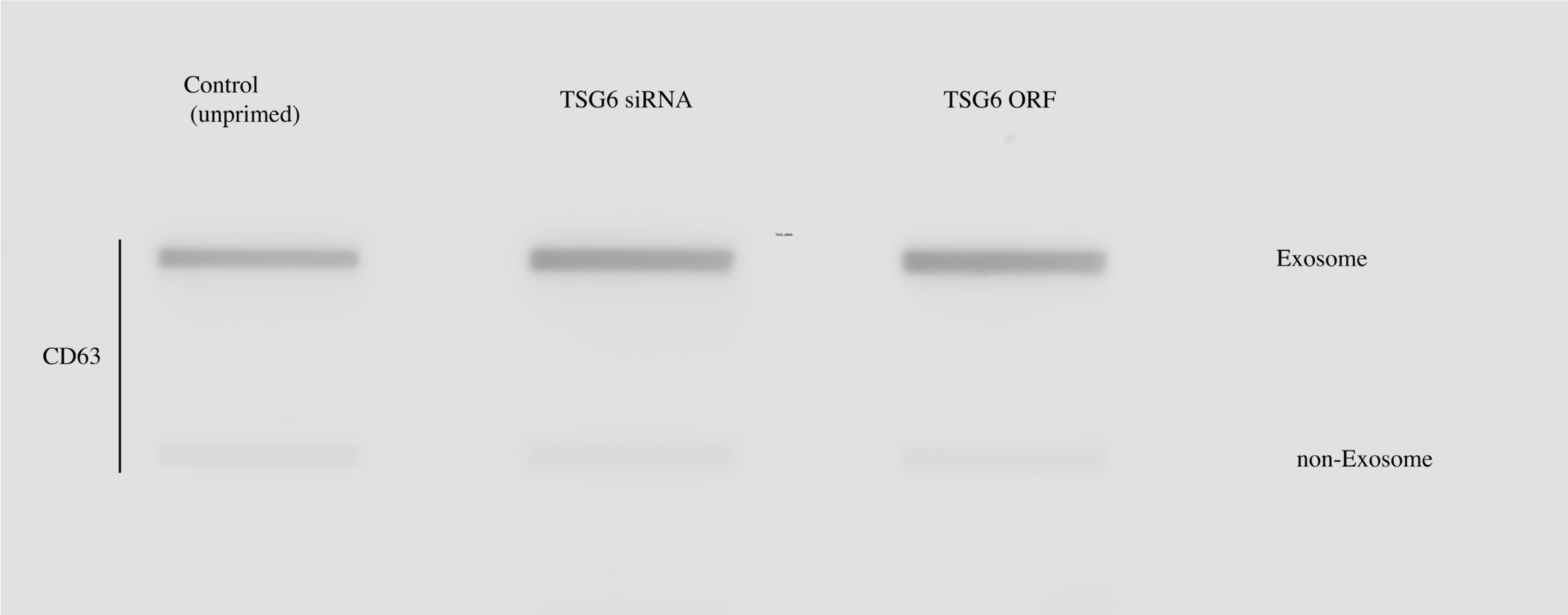
Control
(unprimed)
TSG6 siRNA
TSG6 ORF
CD63
Exosome
non-Exosome

Sup Figure 2C

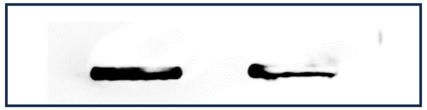

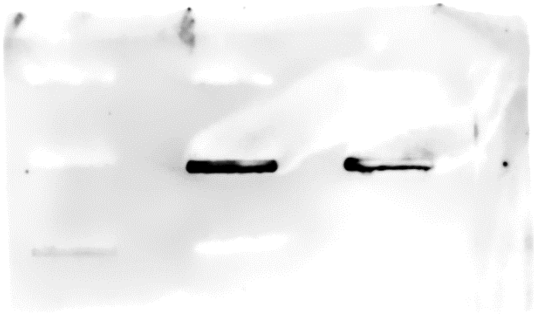

Cell lysates
